# Whole-genome sequencing reveals high genetic diversity of *Streptococcus uberis* isolated from cows with mastitis

**DOI:** 10.1186/s12917-021-03031-4

**Published:** 2021-10-07

**Authors:** Nathália Cristina Cirone Silva, Yongqiang Yang, Marjory Xavier Rodrigues, Tiago Tomazi, Rodrigo Carvalho Bicalho

**Affiliations:** 1 Department of Population Medicine and Diagnostic Sciences, College of Veterinary Medicine, Cornell University, Ithaca, New York USA; 2 Department of Food Science and Nutrition, Faculty of Food Engineering (FEA) , University of Campinas (UNICAMP), Rua Monteiro Lobato, 80, SP 13083-862 Campinas, Brazil; 3 Department of Microbiology, Zhongshan School of Medicine, Sun Yat-sen University, 510080 Guangzhou, China; 4 School of Pharmaceutical Sciences (Shenzhen), Sun Yat-sen University, 510006 Guangzhou, China

**Keywords:** New generation sequencing, Virulence genes, Phylogenetic tree

## Abstract

**Background:**

Bovine mastitis is an important cause of economic loss in dairy farms. *Streptococcus uberis* is among the most frequently isolated bacterial species isolated from cows with mastitis. The aim of this study was to perform an in-depth genetic assessment of *S. uberis* strains isolated from bovine clinical mastitis (CM) and to perform a phylogenetic analysis to represent the evolutionary relationship among *S. uberis* sequences.

**Results:**

A total of 159 isolates was genetically characterized using whole genome sequencing. According to the virulence determinants, all strains harbored the *hasC, leu*S, *per*R, *pur*H, and *pur*N virulence genes. Thirty-four resistance genes were identified in at least one strain. In terms of acquired genes, we observed that 152 (95.6 %) strains had a resistance gene to lincosamine (*lnu*D), 48 (30.2 %) to tetracycline (*tet*M), 4 (2.51 %) to tobramicine (*ant*6), and 1 to lincosamide (*lsa*(E)). MLST detected the Sequence Type (ST)797 (*n* = 23), while 85.5 % of the strains did not match to known STs.

**Conclusions:**

Then, eleven distinct ST were identified after we submitted the new alleles to assign new STs. The other prevalent STs observed were ST1215 (*n* = 58), ST1219 (*n* = 35), and ST1213 (*n* = 15). And it was not possible to identify the MLST of four strains. Phylogenetic lineages indicated a high genomic diversity of *S. uberis* in our collection, confirming that most strains isolated from bovine mastitis have different reservoirs, typical of environmental pathogens.

## Background

Bovine mastitis is one of the major concerns for the dairy industry being associated with direct and indirect economic losses. Direct losses include the increased costs with veterinary services, milk discard, mortality and culling of animals, and reduction of milk quality. On the other hand, indirect losses are considered the most substantial one, which include the reduction of milk production, changing in milk composition, pre-term drying-off, impairment of reproductive performance, animal welfare aspects, and other associated health issues [1, 2].

Many microbial species were described as the cause of bovine mastitis and among them, *Streptococcus* spp. is among the most isolated genera in dairy herds, being associated with both clinical and subclinical forms of the disease [3, 4]. Within the *Streptococcus* genus, *Streptococcus uberis* is the most prevalent species envolving with bovine mastitis [5]. *S. uberis* is a Gram-positive pathogen inducing both clinical and subclinical mastitis, causing reduction of milk production, changes in milk composition and increase of somatic cell count (SCC) in dairy cows [6–8]. The high polymorphism of strains isolated from bovine mastitis indicates that the environment (e.g. bedding used in housing facilities and pastures) is the main reservoir of *S. uberis* [9]. However, recent studies have shown evidences that certain strains might be transmitted from cow to cow during milking [10, 11]. *S. uberis* was also associated with persistent intramammary infections, which could be related to its ability to internalize in the mammary gland [12], along with its increased resistance to antimicrobials [13, 14]. Despite several studies evaluating *S. uberis* have been published in recent years, the role of this species in the epidemiology of mastitis is not completely understood. With the advent of powerful molecular methods, such as whole genome sequencing, it is now possible to detect genetic antimicrobial resistance determinants and virulence factor genes [15]. The advance of knowledge about the genetic features associated with *S. uberis* causing mastitis associated with clinical outcomes such as cure after antimicrobial treatment, death/culling due mastitis, mammary quarter loss and disease reoccurrence, can contribute to the developing of efficient strategies for prevention and control of this pathogen in dairy herds.

The aim of this study was to perform an in-depth genetic assessment of *S. uberis* isolated from bovine clinical mastitis (CM) and to perform a phylogenetic analysis to represent the evolutionary relationship between *S. uberis* sequences.

## Results and discussion

### Descriptive data

A total of 159 *S. uberis* strains were selected from 151 cows. Isolates identified from the same cows were isolated from clinical mastitis occurred in different mammary quarters. Cows from which the strains were isolated had an average number of lactation of 2.6 (SD = 1.4) and DIM of 119.7 (SD = 89.8). In total, 83 % of the isolates (*n* = 132) were recovered from mild cases (i.e., only changes in the milk appearance) of clinical mastitis, while 17 % where either moderate (i.e., changes in milk appearance associated with inflammatory symptoms in the udder) or severe (i.e., changes in the milk and udder associated with systemic inflammatory symptoms).

According to cow-level records, the following outcomes were recorded from cows after CM caused by the *S. uberis* isolates selected herein: mortality after CM (19.5 %), bacteriological cure (44.65 %), mammary quarter loss (10.7 %), clinical cure (87.4 %) and reoccurrence of clinical mastitis (23.9 %).

**Fig. 1:**
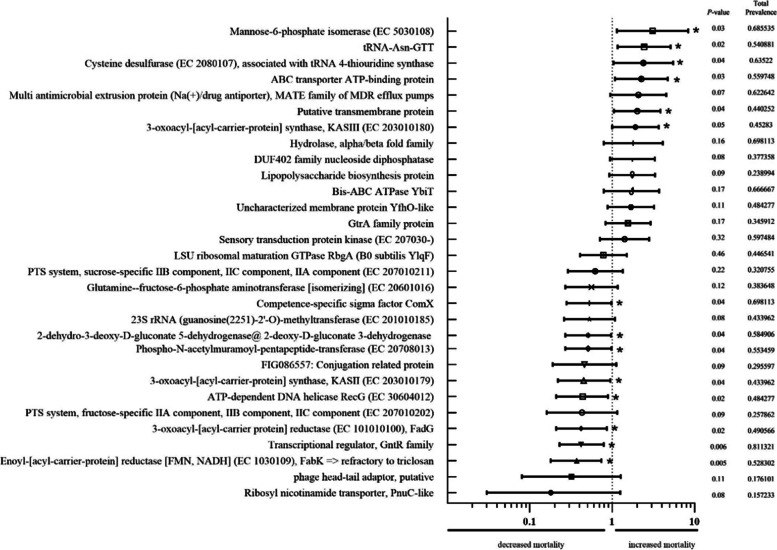
Relative risk for mortality based on gene enconding belonging of bacteria which cause mastitis. * - significant difference

**Fig. 2:**
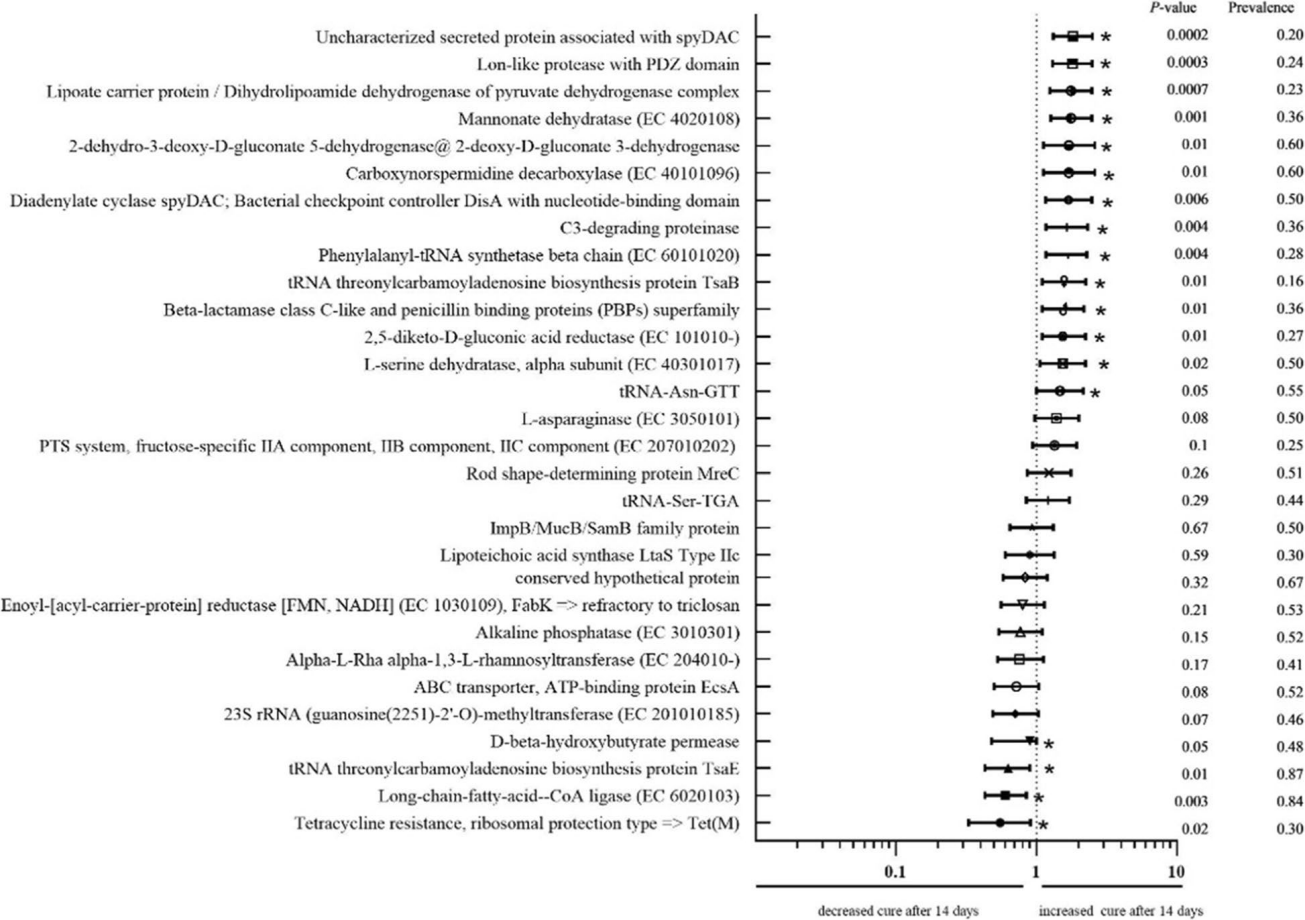
Relative risk for bacteriological cure after 14 days of treatment based on gene encoding belonging of bacteria which cause mastitis. * - significant difference

In Figs. 1 and 2, we demonstrate the relative risk of each encoding gene and the probability of mortality and bacteriological cure after 14 days for cows, respectively. We present the risk, significance, prevalence of positive bacteria and cows infected by them which dye and which alive, and total prevalence of genes in cows.

### Virulence factors

All strains presented five virulence genes: *has*C, *leu*S, *per*R, *pur*H, and *pur*N. They were reported to encode hyaluronic acid capsule (*has*C), Leucyl-tRNA synthetase (*leu*S), peroxide stress regulator (*per*), and are involved with the purine biosynthesis (*pur*H and *pur*N [16–18] (https://www.uniprot.org/uniprot/P67514, www.patricbrc.org). The genes distribution according to the clinical outcome status of cows from which the strains were isolated is presented on Table 1.

**Table 1:** Distribution of virulence factors genes of 159 *Streptococcus uberis* isolated from clinical mastitis according to clinical outcomes recorded during the follow-up period

Virulence factor	Product	Mortality (% of cows infected by positive strains)				Bacteriological cure				Clinical cure				Loss of mammary quarter function.				Reoccurrence of CM				Total strains
		Dead		Alive		No		Yes		No		Yes		No		Yes		No		Yes		
		n	%	n	%	n	%	n	%	n	%	n	%	n	%	n	%	n	%	n	%	
*has*C	UTP–glucose-1-phosphate uridylyltransferase (EC 2.7.7.9)	31	19	128	81	88	55	71	45	20	13	139	87	142	89	17	11	121	76	38	24	159
*leu*S	Leucyl-tRNA synthetase (EC 6.1.1.4)	31	19	128	81	88	55	71	45	20	13	139	87	142	89	17	11	121	76	38	24	159
*per*R	Peroxide stress regulator PerR, FUR family	31	19	128	81	88	55	71	45	0	00	159	100	142	89	17	11	121	76	38	24	159
*pur*H	IMP cyclohydrolase (EC 3.5.4.10) / Phosphoribosylaminoimidazolecarboxamide formyltransferase (EC 2.1.2.3)	31	19	128	81	88	55	71	45	20	13	139	87	142	89	17	11	121	76	38	24	159
*pur*N	Phosphoribosylglycinamide formyltransferase (EC 2.1.2.2)	31	19	128	81	88	55	71	45	20	13	139	87	142	89	17	11	121	76	38	24	159
*cyd*A	Cytochrome d ubiquinol oxidase subunit I (EC 1.10.3.-)	31	20	127	80	87	55	71	45	20	13	138	87	141	89	17	11	120	76	38	24	158
*gid*A	tRNA-5-carboxymethylaminomethyl-2-thiouridine(34) synthesis protein MnmG	31	20	127	80	88	56	70	44	20	13	138	87	141	89	17	11	120	76	38	24	158
*pur*B	Adenylosuccinate lyase (EC 4.3.2.2) @ SAICAR lyase (EC 4.3.2.2)	30	19	127	81	86	55	71	45	20	13	137	87	140	89	17	11	119	76	38	24	157
SP_0121	Ribonuclease J1 (endonuclease and 5’ exonuclease)	31	20	125	80	87	56	69	44	20	13	136	87	139	89	17	11	118	76	38	24	156
SP_0494	CTP synthase (EC 6.3.4.2)	31	20	125	80	86	55	70	45	20	13	136	87	139	89	17	11	118	76	38	24	156
*fba*	Fructose-bisphosphate aldolase class II (EC 4.1.2.13)	31	20	124	80	85	55	70	45	20	13	135	87	138	89	17	11	117	75	38	25	155
*gln*A	Glutamine synthetase type I (EC 6.3.1.2)	30	19	125	81	86	55	69	45	20	13	135	87	138	89	17	11	117	75	38	25	155
r*po*E	DNA-directed RNA polymerase delta subunit (EC 2.7.7.6)	31	20	124	80	85	55	70	45	20	13	135	87	138	89	17	11	117	75	38	25	155
*atm*B	Methionine ABC transporter substrate-binding protein	29	19	123	81	83	55	69	45	20	13	132	87	135	89	17	11	114	75	38	25	152
*ccp*A	Catabolite control protein A	30	20	122	80	82	54	70	46	20	13	132	87	135	89	17	11	114	75	38	25	152
*lux*S	S-ribosylhomocysteine lyase (EC 4.4.1.21) @ Autoinducer-2 production protein LuxS	30	20	122	80	84	55	68	45	20	13	132	87	135	89	17	11	114	75	38	25	152
SP_0320	2-dehydro-3-deoxy-D-gluconate 5-dehydrogenase (EC 1.1.1.127) @ 2-deoxy-D-gluconate 3-dehydrogenase (EC 1.1.1.125)	29	19	123	81	85	56	67	44	10	07	142	93	135	89	17	11	114	75	38	25	152
*clp*P	ATP-dependent Clp protease proteolytic subunit ClpP (EC 3.4.21.92)	30	20	121	80	83	55	68	45	20	13	131	87	134	89	17	11	113	75	38	25	151
Spy_1633		29	19	122	81	83	55	68	45	20	13	131	87	134	89	17	11	113	75	38	25	151
vick	Histidine kinase	29	19	120	81	81	54	68	46	20	13	129	87	132	89	17	11	111	74	38	26	149
SP_2086	Phosphate ABC transporter, permease protein PstA (TC 3.A.1.7.1)	29	20	119	80	81	55	67	45	20	14	128	86	131	89	17	11	110	74	38	26	148
SP_0095	Rhodanese domain protein UPF0176, Firmicutes subgroup	30	21	115	79	82	57	63	43	20	14	125	86	128	88	17	12	107	74	38	26	145
*lep*A	Translation elongation factor LepA	30	21	114	79	79	55	65	45	20	14	124	86	130	90	14	10	107	74	37	26	144
*sod*A	Superoxide dismutase [Mn] (EC 1.15.1.1)	30	21	114	79	81	56	63	44	20	14	124	86	127	88	17	12	106	74	38	26	144
*cps*Y	Methionine biosynthesis and transport regulator MtaR, LysR family	30	21	110	79	79	56	61	44	20	14	120	86	126	90	14	10	103	74	37	26	140
SP_0829	Phosphopentomutase (EC 5.4.2.7)	30	21	110	79	79	56	61	44	20	14	120	86	126	90	14	10	103	74	37	26	140
*cia*R	Two component system response regulator CiaR	29	21	110	79	78	56	61	44	19	14	120	86	123	88	16	12	103	74	36	26	139
SP_0916	Arginine decarboxylase (EC 4.1.1.19)	30	22	109	78	79	57	60	43	20	14	119	86	124	89	15	11	102	73	37	27	139
*gua*A	GMP synthase [glutamine-hydrolyzing], amidotransferase subunit (EC 6.3.5.2) / GMP synthase [glutamine-hydrolyzing], ATP pyrophosphatase subunit (EC 6.3.5.2)	28	20	110	80	77	56	61	44	19	14	119	86	123	89	15	11	102	74	36	26	138
SP_1970	Aspartate–ammonia ligase (EC 6.3.1.1)	30	22	108	78	78	57	60	43	20	14	118	86	124	90	14	10	102	74	36	26	138
SP_1396	Phosphate ABC transporter, ATP-binding protein PstB (TC 3.A.1.7.1)	28	21	108	79	76	56	60	44	19	14	117	86	120	88	16	12	101	74	35	26	136
SP_1398	Phosphate ABC transporter, permease protein PstA (TC 3.A.1.7.1)	28	21	108	79	76	56	60	44	19	14	117	86	120	88	16	12	101	74	35	26	136
SP_0856	Branched-chain amino acid aminotransferase (EC 2.6.1.42)	28	21	107	79	76	56	59	44	18	13	117	87	121	90	14	10	100	74	35	26	135
SP_1780	Oligoendopeptidase F-like protein	4	22	14	78	12	67	6	33	3	17	15	83	15	83	3	17	13	72	5	28	18
cps4L	UDP-N-acetyl-L-fucosamine synthase (EC 5.1.3.28)	3	30	7	70	4	40	6	60	1	10	9	90	9	90	1	10	8	80	2	20	10
SpyM3_0013	Cationic amino acid transporter - APC Superfamily	1	11	8	89	5	56	4	44	1	11	8	89	8	89	1	11	8	89	1	11	9
*psa*C	ABC transporter membrane-spanning permease-manganese transport	0	00	6	100	2	33	4	67	1	17	5	83	5	83	1	17	5	83	1	17	6
*hup*A	Putative DNA-binding protein HU-beta (ACLAME 290)	0	00	2	100	1	50	1	50	20	100	1	50	2	100	0	00	1	50	1	50	2
pbp1A	DD-transpeptidase	0	00	2	100	2	100	0	00	0	00	2	100	2	100	0	00	1	50	1	50	2
*pol*C	DNA polymerase III PolC-type	1	50	1	50	2	100	0	00	0	00	2	100	2	100	0	00	2	100	0	00	2
fbp54	Fibronectin/fibrinogen-binding protein	0	00	1	100	0	00	1	100	0	00	1	100	1	100	0	00	1	100	0	00	1
*gln*P	Glutamine ABC transporter, substrate-binding protein GlnH / Glutamine ABC transporter, substrate-binding protein GlnH / Glutamine ABC transporter, permease protein GlnP	1	100	0	00	1	100	0	00	1	100	0	00	1	100	0	00	0	00	1	100	1
*has*A	Hyaluronan synthase	0	00	1	100	1	100	0	00	0	00	1	100	1	100	0	00	1	100	0	00	1
*neu*B	Putative N-acetylneuraminic acid synthase	0	00	1	100	1	100	0	00	0	00	1	100	0	00	1	100	1	100	0	00	1
*nox*	NADH oxidase	0	00	1	100	0	00	1	100	0	00	1	100	1	100	0	00	1	100	0	00	1
*pep*C	Aminopeptidase C (EC 3.4.22.40)	0	00	1	100	0	00	1	100	20	200	1	100	1	100	0	00	1	100	0	00	1
*pur*L	Phosphoribosylformylglycinamidine synthase, synthetase subunit (EC 6.3.5.3)	0	00	1	100	1	100	0	00	0	00	1	100	1	100	0	00	1	100	0	00	1
SP_0842		0	00	1	100	1	100	0	00	0	00	1	100	0	00	1	100	1	100	0	00	1
SP_1399	Phosphate ABC transporter, permease protein PstC (TC 3.A.1.7.1)	0	00	1	100	1	100	0	00	0	00	1	100	1	100	0	00	1	100	0	00	1

**Fig. 3:**
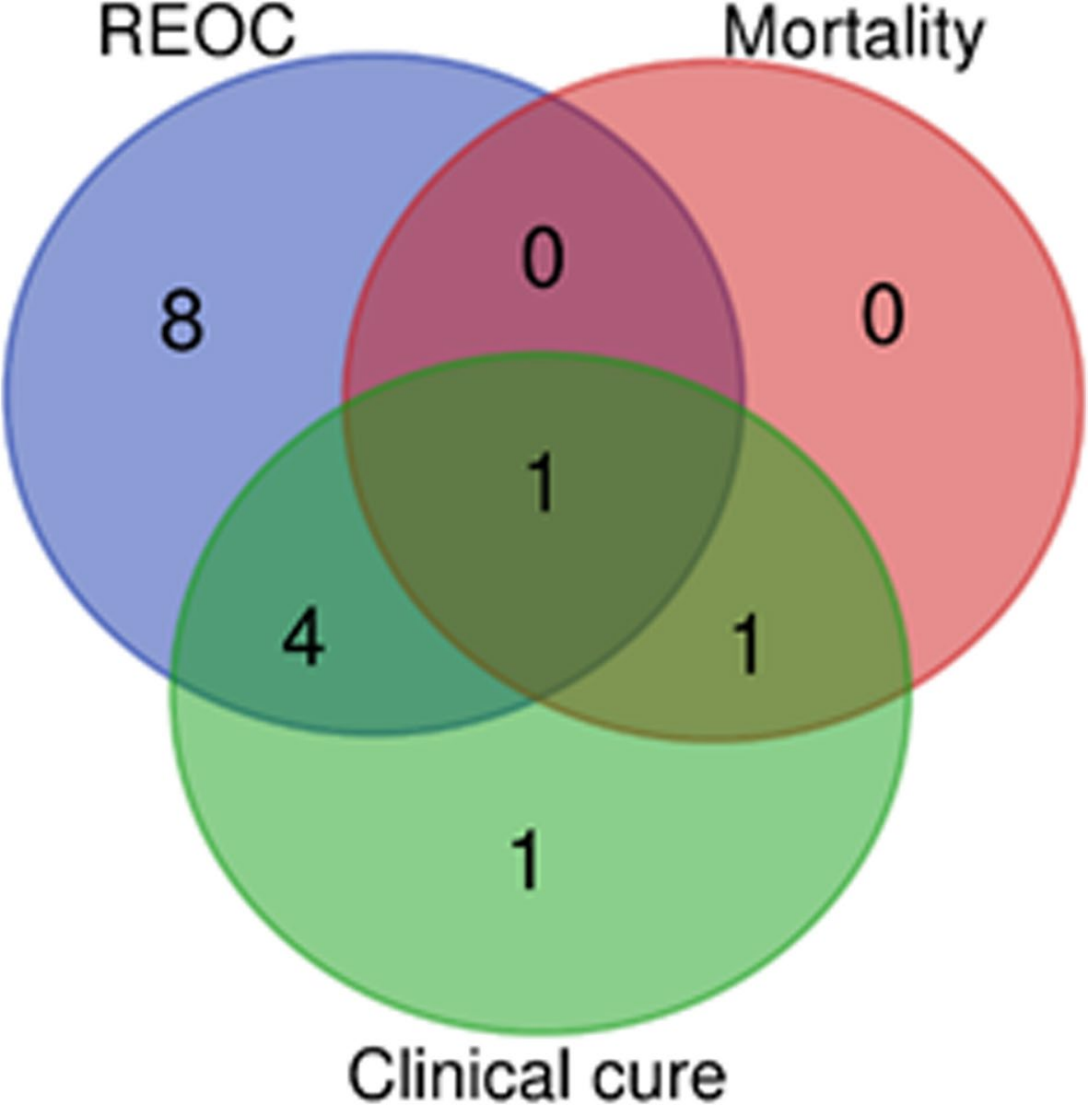
Venn diagram presenting the frequency of virulence genes of *Streptococcus uberis* isolated from clinical mastitis that were associated with the clinical outcomes recorded during the follow-up period. REOC: reoccurrence of CM

Some genes had low prevalence (less than three cows infected by the isolates positives for each gene). Among the genes statistically associated with reoccurrence of CM were *atm*B, *ccp*A, *clp*P, *cps*Y, *lux*S, *sod*A, SP_0095, SP_0320, SP_0829, SP_0916, SP_2086, Spy_1633, and vicK. The genes SP_1970 and SP_0916 were associated with death/culling of the cow during lactation. For clinical cure, *cps*Y, *lep*A, *sod*A, SP_0095, SP_0829, SP_0916, SP_1970 were associated with no cure. No virulence genes were associated with the bacteriological cure. Only the gene SP_0916 was associated with more than two outcomes (risk of reoccurrence, clinical cure and death/mortality). The genes *sod*A, *cps*Y, SP_0829, SP_0095 were associated with reoccurrence and clinical cure. Finally, the gene SP_1970 was associated with mortality and clinical cure (Fig. 3). The Venn diagram (Fig. 3) for virulence, resistance genes and drug target genes was done with the genes that showed statistical significance (*P* < 0.05) for any clinical outcome.

*S. uberis* has several virulence genes that have an important role in its pathogenicity. Among them we can highlight the hyaluronic acid capsule genes (*has*A, *has*B, and *has*C), the plasminogen activator A gene (*pau*A), and the *S. uberis* adhesion molecule gene (*sua*) [16, 19, 20]. In our study, all strains presented hasC, and just one *has*A.

The putative membrane-bound protein transports essential amino acids across the cytoplasmic membrane and it is a virulence factor that promote growth of bacteria in milk [21]. The *vru* cluster co-ordinate the expression of many putative virulence-associated genes during growth of *S. uberis* in milk [22]. These genes and other genes (*pau*A, *opp*, *mtu*A), singly or as a group, have not been shown to be specifically associated with mastitis; however, there is evidence that certain host-adapted strains of *S. uberis* have enhanced ability to cause clinical mastitis [23–25].

Strains of *S. uberis* isolated from cases of bovine mastitis display variable amounts of hyaluronic acid capsule. Capsule production is dependent of the *has* operon, which consists of the *has*AB gene cluster and *has*C gene [16]. The *has* operon comprises the *has*A (encoding the hyaluronan synthase), *has*B (encoding the UDP-glucose dehydrogenase), and *has*C, which encodes the UDP-glucose pyrophosphorylase [26]. The *has*A gene product is essential for capsule production in *S. uberis* [16]. Some studies have reported that because of the capsule absence, *S. uberis* is able to support the bactericidal effect of bovine neutrophils and induce mastitis in dairy cows [27].

The genes *pur*N e *pur*H, which were identified in 100 % of our isolates, are reported to be involved with the purine biosynthesis. These genes are involved with the de novo purine biosynthetic pathway responsible for the synthesis of inosine monophosphate. Studies showed that mutations in purine biosynthetic genes attenuate virulence in *Brucella abortus*, and it was demonstrated the importance of these genes for growth of several pathogens, as *Salmonella enterica* and *Bacillus anthracis*, in human serum [17, 18].

### Resistance factors

The emergence of drug resistance by bacteria has been associated with the overuse of antibiotics [28]. At the same time, mastitis is recognized as the main cause for antimicrobial use in dairy farms [2, 29, 30]. Although *S. uberis* is one of the most important cause of bovine mastitis in dairy herds [31, 32], the resistome of isolates from cows with mastitis demands further understanding. Herein, thirty-four resistance genes were identified in at least one strain. All strains presented *rlm*A (II), *rpo*B, *rpo*C genes, although no mutation was observed. For acquired resistance genes, we observed that 152 (95.6 %) strains had a resistance gene to lincosamine (*lnu*D), 48 (30.2 %) to tetracycline (*tet*M), 4 (2.51 %) to tobramicine (*ant*6), 1 to lincosamide (*lsa*(E)).

The distribution of the genes according to the clinical outcome following the CM diagnosis is presented in Table 2. The gene *pgs*A, which is reported to be associated with daptomycin resistance when have a mutation [33], significantly decreased the risk of death/culling. A study identified that the substitution in two enzymes involved in the cardiolipin biosynthesis pathway, i.e., CdsA (phosphatidate cytidylyltransferase) and PgsA (CDP-diacylglycerol-glycerol-3-phosphate-3-phosphatidyltransferase), were associated with no production of phosphatidylglycerol and cardiolipin from cell membranes [34]. Furthermore, the risk of mastitis reoccurrence significantly increased with the presence of several genes (*ddl*, *fol*P, *gdp*D, *gld*B, *gyr*A, *gyr*B, *lia*F, *lia*R, *lia*S, *lnu*D, *mur*A).

**Table 2:** Distribution of genes associated with antimicrobial resistance of 159 *Streptococcus uberis* isolated from clinical mastitis according to clinical outcomes recorded during the follow-up period

Resistance factor	Product	Antibiotic class	Mortality (% of cows infected by positive strains)				Bacteriological cure				Clinical cure				Loss of mammary quarter function.				Reoccurrence of CM				Total of positive strains
			Dead		Alive		No		Yes		No		Yes		No		Yes		No		Yes		
			n	%	n	%	n	%	n	%	n	%	n	%	n	%	n	%	n	%	n	%	
*rlm*A(II)	23 S rRNA (guanine(748)-N(1))-methyltransferase	Macrolides, Lincosamides	31	19	28	81	88	55	71	45	20	13	139	87	142	89	17	11	121	76	38	24	159
*rpo*B	DNA-directed RNA polymerase beta subunit	Rifamycins, Peptide antibiotics	31	19	128	81	88	55	71	45	20	13	139	87	142	89	17	11	121	76	38	24	159
*rpo*C	DNA-directed RNA polymerase beta’ subunit	Myxopyronins Corallopyronins, Peptide antibiotics	31	19	128	81	88	55	71	45	20	13	139	87	142	89	17	11	121	76	38	24	159
S10p	SSU ribosomal protein S10p	Tetracyclines, Glycylcyclines	30	19	128	81	87	55	71	45	20	13	138	87	141	89	17	11	120	76	38	24	158
*alr*	Alanine racemase	Cycloserine	31	20	126	80	86	55	71	45	20	13	137	87	140	89	17	11	119	76	38	24	157
EF-G	Translation elongation factor G	Fusidic acid	31	20	126	80	87	55	70	45	20	13	137	87	140	89	17	11	119	76	38	24	157
S12p	SSU ribosomal protein S12p	Aminoglycosides	31	20	126	80	87	55	70	45	20	13	137	87	140	89	17	11	119	76	38	24	157
*pg*sA	CDP-diacylglycerol–glycerol-3-phosphate 3-phosphatidyltransferase	Peptide antibiotics (daptomycin)	28	18	127	82	85	55	70	45	20	13	135	87	138	89	17	11	121	78	34	22	155
*fab*K	Enoyl-[acyl-carrier-protein] reductase [FMN, NADH]	Triclosan	30	19	124	81	84	55	70	45	20	13	134	87	138	90	16	10	116	75	38	25	154
*kas*A	3-oxoacyl-[acyl-carrier-protein] synthase, KASII	Isoniazid, Triclosan	30	19	124	81	84	55	70	45	20	13	134	87	138	90	16	10	116	75	38	25	154
D	Lincosamide nucleotidyltransferase = > Lnu(D)	Lincosamides	30	20	122	80	83	55	69	45	20	13	132	87	135	89	17	11	114	75	38	25	152
*lia*F	Membrane protein LiaF(VraT), specific inhibitor of LiaRS(VraRS) signaling pathway	Peptide antibiotics (daptomycin)	30	20	121	80	84	56	67	44	20	13	131	87	134	89	17	11	113	75	38	25	151
*Lia*R	Cell envelope stress response system LiaFSR, response regulator LiaR(VraR)	Peptide antibiotics (daptomycin)	30	20	121	80	84	56	67	44	20	13	131	87	134	89	17	11	113	75	38	25	151
*Lia*S	Cell envelope stress response system LiaFSR, sensor histidine kinase LiaS(VraS)	Peptide antibiotics (daptomycin)	30	20	121	80	84	56	67	44	20	13	131	87	134	89	17	11	113	75	38	25	151
*mur*A	UDP-N-acetylglucosamine 1-carboxyvinyltransferase	Fosfomycin	30	20	121	80	84	56	67	44	20	13	131	87	134	89	17	11	113	75	38	25	151
*gid*B	16 S rRNA (guanine(527)-N(7))-methyltransferase	Aminoglycosides	29	19	121	81	83	55	67	45	20	13	130	87	133	89	17	11	112	75	38	25	150
GdpD	Glycerophosphoryl diester phosphodiesterase	Peptide antibiotics (daptomycin)	30	20	119	80	83	56	66	44	20	13	129	87	132	89	17	11	111	74	38	26	149
*fol*P	Dihydropteroate synthase	Sulfonamides	30	20	117	80	84	57	63	43	20	14	127	86	131	89	16	11	109	74	38	26	147
*ddl*	D-alanine–D-alanine ligase	Cycloserine	29	20	117	80	81	55	65	45	20	14	126	86	129	88	17	12	108	74	38	26	146
Iso-tRNA	Isoleucyl-tRNA synthetase	Mupirocin	30	21	116	79	83	57	63	43	20	14	126	86	130	89	16	11	109	75	37	25	146
*fol*A, Dfr	Dihydrofolate reductase	Diaminopyrimidines	29	20	115	80	81	56	63	44	20	14	124	86	128	89	16	11	107	74	37	26	144
*gyr*B	DNA gyrase subunit B	Novobiocin	29	20	115	80	80	56	64	44	20	14	124	86	127	88	17	12	106	74	38	26	144
*gyr*A	DNA gyrase subunit A	Ciprofloxacin	30	21	113	79	80	56	63	44	20	14	123	86	127	89	16	11	106	74	37	26	143
EF-Tu	Translation elongation factor Tu	Elfamycins	29	21	112	79	77	55	64	45	20	14	121	86	127	90	14	10	105	74	36	26	141
*tet*M	Tetracycline resistance, ribosomal protection type = > Tet(M)	Tetracyclines	6	13	42	88	20	42	28	58	6	13	42	88	43	90	5	10	39	81	9	19	48
*par*C	DNA topoisomerase IV subunit A	Ciprofloxacin	0	00	7	100	4	57	3	43	2	29	5	71	6	86	1	14	7	100	0	00	7
*ant* (6)-I	Aminoglycoside 6-nucleotidyltransferase	Aminoglycosides	1	25	3	75	1	25	3	75	0	00	4	100	4	100	0	00	4	100	0	00	4
*bce*R	Two-component response regulator BceR	Peptide antibiotics (bacitracin)	1	50	1	50	1	50	1	50	0	00	2	100	2	100	0	00	1	50	1	50	2
*lsa*(E)	ABC-F type ribosomal protection protein	Lincosamides	0	00	1	100	0	00	1	100	0	00	1	100	1	100	0	00	1	100	0	00	1

Previous studies have reported the resistance of *S. uberis* and they demonstrated that it is higher than *S. dysgalactiae*, which is another important *Streptococcus* causing mastitis in dairy cows [14, 35]. Despite the importance of bacterial resistance, few studies assessed the association between antimicrobial resistance genes of mastitis-causing streptococci and clinical outcomes after intramammary infections [15].

A total of 95.6 % of isolates enrolled in our study presented the gene *lnu*D, although it was not associated with clinical outcomes of cows affected with clinical mastitis. The gene *lnu*D was reported to be associated with resistance to lincomycin [36]. The mechanism of action of lincosamides, including lincomycin, is to prevent protein synthesis by inhibiting the peptidyltransferase to several nucleotides of 23 S rRNA in the 50 S subunit of the bacterial ribosome. Along with the gene *lnu*D, other genes were reported to confer to streptococci resistance to lincosamides, such as *lnu*B and *lin*B [15, 37, 38]. In our study only two strains presented the *Inu*B and none had the *linB* gene.

The gene *lsa*(E), identified in one of our strains, was also reported to confer resistance to lincosamide besides streptogramin A and pleuromutilin antibiotics [39]. This gene was also identified in *Enterococcus faecalis* and *Staphylococcus aureus* strains, and were reported be located on a multiresistance gene cluster, suggesting that the intra- and inter-genus dissemination and exchange of resistance genes could occur via the plasmids [40]. Therefore, monitoring this gene in gram-positive pathogens causing mastitis, such as *S. uberis* can be relevant to prevent resistance to antimicrobials used to treat mastitis [40].

Herein, the gene *tet*M, which is associated with resistance to tetracycline, decreased the risk of bacteriological cure after 14 days of CM diagnosis. In total, 30.2 % of all enrolled strains had the *tet*M gene. Recent studies have reported low *in vitro* susceptibility of *S. uberis* to tetracycline [13, 41], which may be attributed to the excessive use of these antimicrobials by the systemic route for treatment of infections in dairy cows and as growth promoter in other species. However, we were not able to indicate any plausible explanation why cows with CM caused by *S. uberis* having the *tet*M gene had lower risk of bacteriological cure, especially because tetracycline was not used for CM treatment on the selected herd. Furthermore, this antimicrobial is not labeled for treatment of bovine mastitis in US. Tetracycline is an antimicrobial frequently used systemically to treat respiratory and hoof infection in cattle [30], which may explain the presence of resistant *S. uberis* strains among our bacteria collection.

Four strains presented the gene *ant*6 which confers resistance to a tobramycin, an aminoglycoside with a broad antibacterial spectrum *in vitro*, and pharmacokinetic properties similar to gentamicin [42]. The resistance to aminoglycosides has clinical importance, since combination of penicillin G with an aminoglycoside has been recommended for severely ill patients [43]. In addition, penicillin-based products are among the antimicrobials approved to be used for treatment of mastitis in US [44].

The use of antibiotics in food-producing animals can promote the bacterial resistance and allow the presence of antibiotic residues in derived products from animals consumed by human [45].

### MLST

Multi Locus Sequence Type (MLST) is a technique used to analyze constitutive genes based on single nucleotide polymorphism (SNP) (https://pubmlst.org/bigsdb?db=pubmlst_suberis_seqdef).

High genetic diversity was observed in our collection of isolates, which was reflected by a large number of sequence types (STs). In total, eleven distinct STs were observed in our study. Twenty-three strains were classified ST797, which is the only known sequence type among our isolates. Of those, 8 (34.8 %) had bacteriological cure, and 6 (26.1 %) died or were culled during the follow up period. The remained 136 strains did not match with any ST and received a new number.

In addition to the ST797, the most identified MLST-types were ST1215 (*n* = 58) and ST1219 (*n* = 35). ST1215 was isolated from 58 cows, of which 30 (54.5 %) had bacteriological cure, 8 (14.5 %) died or were culled, and 5 (8.6 %) lost the functionality of the affected mammary quarter. Of the 35 isolates identified as ST1219, 13 (37.1 %) had bacteriological cure, 6 (17.1 %) died or were culled. Other types identified from our bacteria collection were ST1213 (*n* = 15 isolates), ST1216 (*n* = 8), ST1221 (*n* = 7), ST1214 (*n* = 2), ST1217 (*n* = 2), ST1218 (*n* = 2), ST1220 (*n* = 2) and ST1212 (*n* = 1). It was not possible to identify the MLST of four strains.

The STs found did not belong to any clonal complex (https://pubmlst.org/bigsdb?db=pubmlst_suberis_seqdef). The most commons MLST types found were ST1215, ST1219, and ST797. Among them, the cows infected by ST797 showed higher prevalence of death, whereas cows isolated with the ST1215 had higher prevalence of bacteriological cure.

The route of transmission of *S. uberis* has been discussed. Various aspects are related with the routes of transmission, being important to consider the interaction host-pathogen and infection pressure [10]. Although *S. uberis* is one of the main pathogens causing mastitis, its epidemiology is not totally understood. The understanding of epidemiological aspect associated with mastitis-causing *S. uberis* can help in the development of focused strategies to control this pathogen in dairy farms.

In our study, we observed 10 new STs and one already known. Davies et al., 2016 reported 195 different STs of *S. uberis* in 52 herds. Only in 10 herds, eleven or more sequence types per herd were observed, showing that in general few STs are related with mastitis within a herd. 71 % of cows were infected by the three more prevalent STs (ST1215, ST1219 and ST797), suggesting that specific strains are more likely to cause mastitis than others are. Although the transmission of *S. uberis* occur mainly by the environmental route, the transmission from cow to cow can be facilitated in herds with inadequate practices for prevention of contagious pathogens of mastitis (e.g., poor milking routine) [37].

### Phylogenetic analysis

**Fig. 4:**
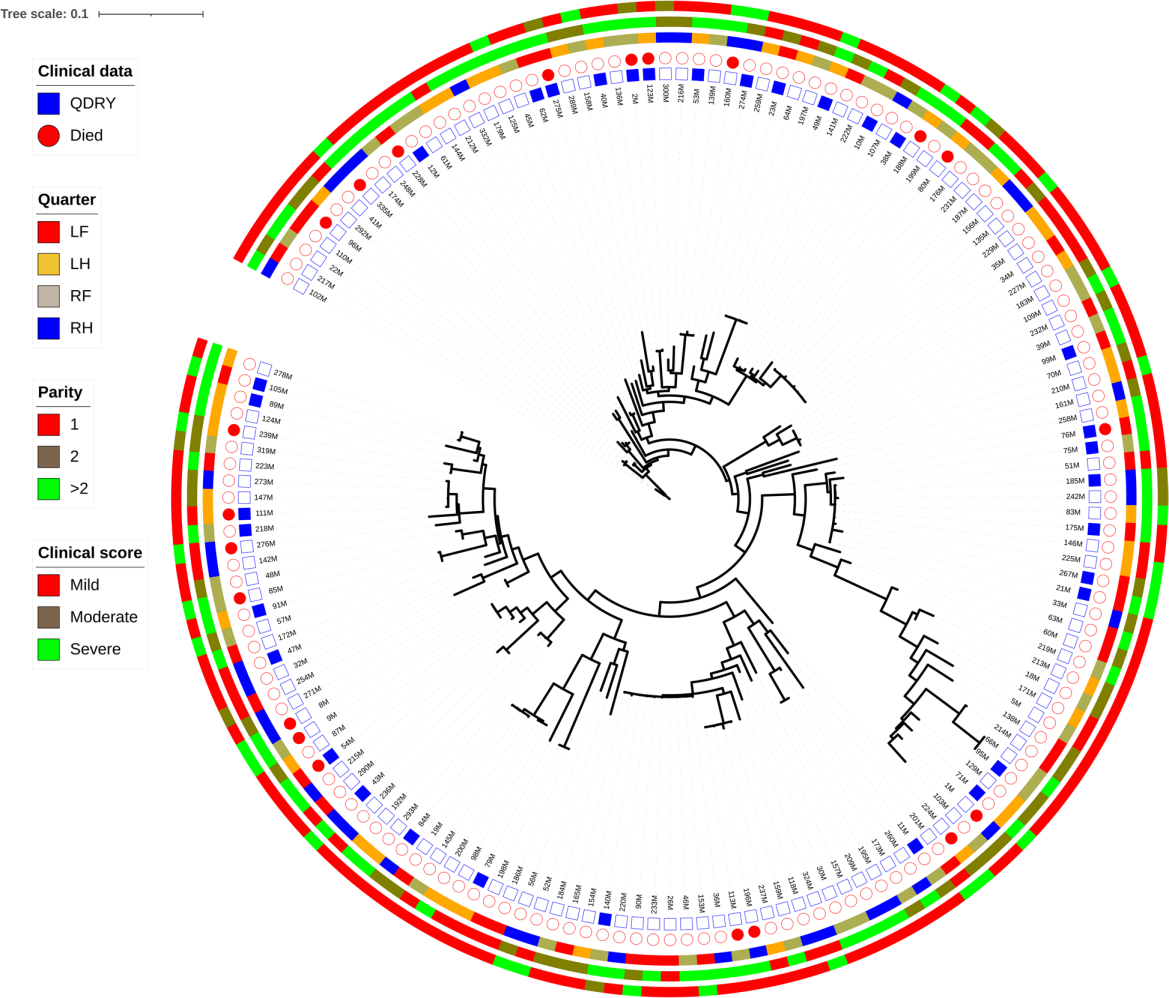
Phylogeny of core genome SNPs in 159 genomes of *Streptococcus uberis* isolates from dairy cows with clinical mastitis and according to the clinical data recorded for each case. The RAxML program was used to calculate the phylogenetic tree to construct a maximum likelihood phylogeny

The pangenome of 6,547 unique protein-coding sequences was performed using 159 *S. uberis* strains enrolled in the study. A total of 29,518 SNPs extracted from the 1,421 core genes was used to infer the ML phylogeny. The results revealed a deep branching and scattered population structure that was broadly classified into distinct phylogenetic lineages, indicating a high genomic diversity of *S. uberis* isolates studied. The prevalence of ST1215 contributed to the emergence of the unique dominant phylogroup. This lineage included strains isolated from all four mammary quarters, mainly associated with mild clinical score of CM, and with cows with more than 2 lactations. However, two cows infected by the isolates within this phylogroup have died or were culled after clinical mastitis caused by *S. uberis*. According to clinical outcomes, the isolates were unclustered and intermingled among strains associating with various clinical responses (Fig. 4).

The phylogenetic tree showed that the strains are diverse and, even when they are considered similar, it could yield similar (e.g., strains 186 M and 198 M) or different clinical outcomes (e.g., strains 95 M and 66 M).

## Conclusions

In the present study, 159 *S. uberis* isolates were obtained from cows with clinical mastitis and their genomes were successfully investigated. Virulence and resistance genes were widely identified among isolates and associated with clinical outcomes. Interestingly, from eleven STs identified only one was previously reported, the other ten new STs were documented through this work. Although the strains were isolated from a single herd, they were highly diverse, which confirms *S. uberis* as an environmental pathogen. Our results can be used as reference for understanding the epidemiology of *S. uberis* causing mastitis, and also, in future studies targeting the development of new strategies for control and prevention of mastitis caused by this pathogen in dairy herds.

## Materials and methods

### Origin of isolates

*S. uberis* were isolated from cases of clinical mastitis identified in a large commercial dairy farm located near Ithaca, New York. The farm milked approximately 4,100 Holstein cows 3 times daily in a 100-stall rotary milking parlor. The animals were housed in freestall barns, with concrete stalls covered with mattresses and bedded with manure solids. The farm had an average milk production per cow of 40.4 Kg (42.2 Kg of energy corrected milk) and bulk milk SCC of 135.330 cells/mL during the period of bacteria isolation.

Farm personnel recorded the severity scores of mastitis as mild (changes in the milk appearance), moderate (changes in the milk appearance associated with udder edema) or severe (presence of systemic signs such as fever, dehydration, prostration) and the distribution of scores was extracted from the farm management software (Dairy Comp 305; Valley Agricultural Software, Tulare, CA).

Strains were identified in a contemporary clinical trial evaluating the efficacy of four protocols for treatment of clinical mastitis caused by Gram-positive pathogens [46]. Briefly, all clinical mastitis cases identified on the farm had two milk samples collected using aseptic methods. One milk sample was collected by the herdsmen as part of the farm’s selective treatment program of CM, which was submitted for aerobic culture using the guidelines of *National Mastitis Council* (2017). The second milk sample was collected by the researchers and cultured only if the first sample had identification of *S. uberis.* In this step, analysis of total Gram-positive bacteria count was performed using the technique of Agar droplets [47] using a selective and differential culture medium (Accutreat®, FERA Diagnostics and Biologicals,, Ithaca, NY). Results of bacterial counts are published elsewhere [46]. A single colony was selected from the aforementioned culture plate and streaked onto a CHROMagar Streptococcus base (CHROMagar, France) plate followed by incubation overnight at 37 °C. This procedure was repeated two more times to ensure purity and a single colony was used for further analysis. The strains used in this study were isolated during the aforementioned procedure using the milk samples collected before CM treatment.

The cows were treated with antibiotics within 48 h after CM identification. As our study was performed contemporarily with another study [48], cows identified with Gram-positive mastitis were randomly allocated to three treatment groups: amoxicillin (label use), 3 infusions with 62.5 mg of amoxicillin (Amoxi-Mast, Merck Animal Health, Millsboro, DE) performed at 12 ± 2 h apart; amoxicillin (extra-label), 5 infusions once a day with 62.5 mg of amoxicillin (Amoxi-Mast, Merck Animal Health); or ceftiofur (label use), 5 infusions once a day with 125 mg of ceftiofur hydrochloride (Spectramast, Zoetis, Kalamazoo, MI) [46].

Follow up outcomes for cows from which the *S. uberis* strains were originated were registered using the dataset of Tomazi et al. [48]. Briefly, clinical cure was defined as the return of milk to normal appearance according to a clinical examination performed 14 ± 3 days after CM diagnosis. Bacteriological cure was defined as the absence of bacterial growth in milk cultures performed at 14 ± 3 days after CM diagnosis. A recurrent case of CM was defined when a new case occurred in the same quarter from 15 to 90 d after identification of CM and the milk culture yielded the same bacterial species isolated at diagnosis. A quarter loss was defined as the loss of mammary quarter physiological function due to the damage caused by the mastitis case. And the culling or death of cows was based on the farm records up to 90 days of CM diagnosis [46].

### Bacterial identification

The DNA was extracted from each bacterial isolate using DNAasy Power food Microbial Kit (Qiagen, Valencia, CA, USA) following the manufacturer’s instructions. NanoDrop ND-1000 spectrophotometer (NanoDrop Technologies, Rockland, DE) was used for DNA quantification. Then, a PCR for the 16 S ribosomal DNA gene amplification was performed using a mix constituted of: 10 pmol of each fD1 forward and rP2 reverse primers [48], Econo-Taq Plus Green 1× Master Mix (Lucigen, Middleton, WI), 280 to 350 ng of template DNA, and ultrapure distilled water (added to complete the volume to 100 µL). The parameters used for amplification were 94 °C for 5 min, 57 °C for 2 min, and 72 °C for 2 min followed by 29 cycles of 94 °C for 2 min, 57 °C for 30 s, and 72 °C for 2 min, with a final extension of 72 °C for 10 min [49]. The presence of PCR products was confirmed by agarose gel electrophoresis (1.2 % wt/vol) with 0.5 µg/mL ethidium bromide. The PCR products were purified using Gel/PCR Fragments Extraction Kit (IBI Scientific, Peosta, IA) following the manufacturer’s recommendations. The purified DNA samples were submitted to the Cornell University Institute of Biotechnology for Sanger sequencing using 8 pmol of primer fD1 and 300 ng of PCR products. For identification of species, we compared our FASTA sequences with the sequences stored in GenBank, using the BLAST algorithm (http://blast.ncbi.nlm.nih.gov/Blast.cgi).

### Whole-genome sequencing

Samples were diluted by adding UltraPure Water (Invitrogen, Waltham, MA) until a concentration of 0.2 ng/µl, measured using a Qubit fluorometer (Thermo Fisher Scientific, Waltham, MA). After normalization, the samples were used as an input to the Nextera XT DNA Sample Prep Kit (Illumina Inc. San Diego, CA). The library preparation was done according to the manufacturer’s protocol (Nextera® DNA Library Prep Reference Guide). Tagmentation of samples was done using 1 ng of template, then PCR amplification was done using a unique combination of barcode primers (provided by manufacture). The purification of libraries was performed using Mag-Bind Totalpure NGS (Omega BioTek - Norcross, GA) bead purification and then normalized through Library Normalization beads/additives. For preparation of cluster generation and sequencing, equal volumes of normalized libraries were combined, diluted in hybridization buffer and heat denatured. Finally, we performed pair-end sequencing using a MiSeq Reagent Kit v3 (600 cycles) in the Illumina MiSeq platform.

#### Genome sequence analyzing

The quality of the original reads was evaluated using FASTQC. The potential contamination of sequences was checked by Kraken2 (Taxonomic sequence classification system) [50].

The sequencing reads were submitted to the comprehensive genome analysis service using Pathosystems Resource Integration Center (PATRIC 3.2.96) [51]. The reads were assembled using SPAdes [52] and the genomes were annotated using the Rast tool kit available in the PATRIC system, as part of the all-bacteria Bioinformatics Resource Center available online [53]. *In silico* multilocus sequence typing (MLST) was performed by MLST 1.8 (https://cge.cbs.dtu.dk/services/MLST/). Acquired antibiotic resistance genes (ARGs) were identified using ABRicate version 0.5 (https://github.com/tseemann/abricate) by aligning genome sequences to the ResFinder database [54]. Virulence genes were identified using VFDB database [55]. Plasmid replicon types were detected using PlasmidFinder v1.3. [56]. The IS elements were confirmed by searching in ISFinder (https://www-isfinder.biotoul.fr).

#### Phylogenetic analysis

For each de novo assembly, coding sequences were predicted using Prodigal v. 2.6 [57] and annotated using the rapid prokaryotic genome annotation tool, Prokka [58]. The core genes were identified and used to build the core genome using Roary [59] with the –e –mafft setting to create a concatenated alignment of core genomic CDS. SNP-sites (https://github.com/sanger-pathogens/snp-sites) was used to extract the core genomic SNPs [60]. To construct a maximum likelihood phylogeny of the sequencing isolates, RAxML was used with the generalized time-reversible model and a GTRGAMMA distribution to model site-specific rate variation [61]. Support for the ML phylogeny was assessed by 100 bootstrap pseudo-analyses of the alignment data. We used iTOL [62] and FigTree (www.tree.bio.ed.ac.uk/software/figtree/) to visualize and edit the phylogenetic tree.

#### Statistical analysis

Descriptive analysis of gene frequency and distribution of genes according to treatment outcomes was performed using JMP PRO 14 (SAS Institute Inc., Cary, NC). Using JMP Pro 14, we selected 30 most important encoding gene through of Predictor Screening and we used 100,000 trees to make the analyzes. We used this to predict the most important genes for variables mortality and bacteriological cure in 14 days. Med Calc was used to calculate risk relative of each variable and prism (GraphPad) was used for plot data.

The Venn diagram was performed using the website: http://bioinformatics.psb.ugent.be/webtools/Venn/ .

## Data Availability

The datasets generated and/or analysed analyzed during the current study are available in database from at www.patricbrc.org and directly from the corresponding author on reasonable request.
